# Mode of Delivery among HIV-Infected Pregnant Women in Philadelphia, 2005-2013

**DOI:** 10.1371/journal.pone.0144592

**Published:** 2015-12-14

**Authors:** Dana R. Thompson, Florence M. Momplaisir, Joëlla W. Adams, Baligh R. Yehia, Emily A. Anderson, Gregg Alleyne, Kathleen A. Brady

**Affiliations:** 1 Center for Women’s and Children’s Health Research, Christiana Care Health Systems, Newark, Delaware, United States of America; 2 Division of Infectious Diseases and HIV Medicine, Drexel University College of Medicine, Philadelphia, Pennsylvania, United States of America; 3 AIDS Activities and Coordinating Office, Philadelphia Department of Public Health, Philadelphia, Pennsylvania, United States of America; 4 Division of Infectious Diseases, University of Pennsylvania Hospital, Philadelphia, Pennsylvania, United States of America; 5 Leonard Davis Institute of Health Economics, University of Pennsylvania, Philadelphia, Pennsylvania, United States of America; 6 Department of Obstetrics and Gynecology, Drexel University College of Medicine, Philadelphia, Pennsylvania, United States of America; University of North Carolina School of Medicine, UNITED STATES

## Abstract

**Objective:**

Current guidelines call for HIV-infected women to deliver via scheduled Caesarean when the maternal HIV viral load (VL) is >1,000 copies/ml. We describe the mode of delivery among HIV-infected women and evaluate adherence to relevant recommendations.

**Study Design:**

We performed a population-based surveillance analysis of HIV-infected pregnant women in Philadelphia from 2005 to 2013, comparing mode of delivery (vaginal, scheduled Caesarean, or emergent Caesarean) by VL during pregnancy, closest to the time of delivery (≤1,000 copies/ml versus an unknown VL or VL >1,000 copies/ml) and associated factors in multivariable analysis.

**Results:**

Our cohort included 824 deliveries from 648 HIV-infected women, of whom 69.4% had a VL ≤1,000 copies/ml and 30.6% lacked a VL or had a VL >1,000 copies/ml during pregnancy, closest to the time of delivery. Mode of delivery varied by VL: 56.6% of births were vaginal, 30.1% scheduled Caesarean, and 13.3% emergent Caesarean when the VL was ≤1,000 copies/ml; when the VL was unknown or >1,000 copies/ml, 32.9% of births were vaginal, 49.9% scheduled Caesarean and 17.5% emergent Caesarean. In multivariable analyses, Hispanic women (adjusted odds ratio (AOR) 0.17, 95% Confidence Interval (CI) 0.04–0.76) and non-Hispanic black women (AOR 0.27, 95% CI 0.10–0.77) were less to likely to deliver via scheduled Caesarean compared to non-Hispanic white women. Women who delivered prior to 38 weeks’ gestation (AOR 0.37, 95% CI 0.18–0.76) were also less likely to deliver via scheduled Caesarean compared to women who delivered after 38 weeks’ gestation. An interaction term for race and gestational age at delivery was significant in multivariable analysis. Non-Hispanic black (AOR 0.06, 95% CI 0.01–0.36) and Hispanic women (AOR 0.03, 95% CI 0.00–0.59) were more likely to deliver prematurely and less likely to deliver via scheduled C-section compared to non-Hispanic white women. Having a previous Caesarean (AOR 27.77, 95% CI 8.94–86.18) increased the odds of scheduled Caesarean delivery.

**Conclusions:**

Only half of deliveries for women with an unknown VL or VL >1,000 copies/ml occurred via scheduled Caesarean. Delivery prior to 38 weeks, particularly among minority women, resulted in a missed opportunity to receive a scheduled Caesarean. However, even when delivering at or after 38 weeks’ gestation, a significant proportion of women did not get a scheduled Caesarean when indicated, suggesting a need for focused public health interventions to increase the proportion of women achieving viral suppression during pregnancy and delivering via scheduled Caesarean when indicated.

## Introduction

The success of preventative interventions has decreased mother-to-child transmission (MTCT) of HIV in industrialized countries to less than 2% [1]. The key to this dramatic decrease is the availability and prescription of antiretroviral therapy (ART) to HIV-infected pregnant women for the purpose of achieving viral suppression before delivery. However, too many women do not achieve viral suppression by delivery [2]. Prior studies indicate that women with a HIV viral load (VL) >1,000 copies/ml at the time of delivery are at greater risk of MTCT of HIV compared to women with a VL ≤1,000 copies/ml. [3]. In these women, delivery via scheduled Caesarean section (C-section) is an effective intervention proven to reduce the risk of HIV transmission. The protective effect of a scheduled C-section before the onset of labor and rupture of membranes was first described in the 1990s by clinical trials comparing scheduled C-section versus usual care in international cohorts [4–5]. Studies conducted in the modern ART era show that delivery via scheduled C-section, rather than vaginal delivery or emergent C-section, most effectively reduces the risk of vertical transmission when maternal VL is elevated near delivery [1].

These findings led the American College of Obstetricians and Gynecologists (ACOG) Committee on Obstetric Practice and the United States Department of Health and Human Services (DHHS) Panel to issue recommendations regarding scheduled C-sections. These guidelines call for HIV-infected women with an VL >1,000 copies/ml to be counseled on the potential benefit of a scheduled C-section to prevent MTCT; receive ART during pregnancy; have a C-section scheduled at 38 weeks' gestation; and receive intravenous zidovudine during labor [6,7]. However, little is known regarding compliance with these guidelines. Potential factors that result in women not delivering via scheduled C-section include lack of engagement in prenatal care, late HIV diagnosis, birth before the scheduled C-section, and patient/provider choice [7–10]. The primary objective of this study was to evaluate adherence to current guidelines recommending a scheduled C-section for HIV-infected women with an unknown VL or VL >1,000 copies/ml for HIV-infected women delivering in Philadelphia from 2005–2013. Additionally, maternal demographic and clinical factors associated with delivery via scheduled C-section for HIV-infected women with an unknown VL or VL >1,000 copies/ml were evaluated through analysis of HIV surveillance data.

## Materials and Methods

### Study Population

We identified all HIV-infected women who delivered a live infant between 2005 and 2013 where the mother was a resident of Philadelphia or the birth occurred in Philadelphia. Multiple gestation deliveries were included as a single observation. Demographic, clinical, and engagement in care information for each mother-infant pair were abstracted from the Philadelphia Enhanced Perinatal Surveillance (EPS) project. EPS is a population-based surveillance system of HIV-infected pregnant women that includes 15 participating areas reporting a high incidence of HIV. The Philadelphia EPS is conducted through the Philadelphia Department of Public Health as a part of the Centers for Disease Control and Prevention's (CDC) surveillance cooperative agreement. The overarching goals of the EPS are to assist in timely evaluation of perinatal prevention efforts, monitor the implementation of the US Public Health Service recommendations for counseling and voluntary testing of pregnant women, and to assess ART use among HIV-infected pregnant women to prevent MTCT [11]. EPS identifies HIV-exposed infants through active case finding, utilizing reports from healthcare providers, delivery hospitals, laboratory reporting, and matching HIV-infected women with vital records. EPS data are obtained from medical chart abstractions (HIV care, prenatal care, intrapartum care, pediatric care records, and vital statistic records) by a trained abstractor. In our study, laboratory values, such as VL during pregnancy, closest to the time of delivery, were determined by merging EPS with the enhanced HIV/AIDS Reporting System (eHARS). eHARS is a surveillance system of all reported HIV/AIDS cases in Pennsylvania; the combined dataset for the state and Philadelphia is maintained by the Pennsylvania and Philadelphia Department of Public Health. The Philadelphia Department of Public Health Institutional Review Board exempted this study from review.

### Variables

#### Outcome Variables

Our primary outcome, mode of delivery, was dichotomously categorized as scheduled C-section versus other modes of delivery (emergent C-section or vaginal delivery). Our definition for a scheduled C-section was based upon that of the DHHS which categorizes a C-section as scheduled if the procedure is done before the onset of labor or rupture of membranes. Emergent C-section was defined as a Caesarean performed after the onset of labor or rupture of membranes. When the mode of delivery was unknown, the case was removed from the analysis (n = 12).

#### Clinical Variables of Interest

Demographic variables included age, race/ethnicity, marital status, health insurance type, HIV risk exposure category, and illicit drug use. Age at delivery was categorized as 16–24, 25–34 and ≥35; race was categorized as non-Hispanic white, non-Hispanic black, and Hispanic; marital status as married and unmarried; and health insurance as public (Medicaid, Medicare, state funded insurance, and other publicly funded insurance), private (private insurance, HMO private insurance, and self-insured), and uninsured. HIV risk exposure was determined using the Centers for Disease Control and Prevention’s hierarchy of reported exposure categories [12]. The HIV risk exposure variable dichotomized risk as either including intravenous drug use or all other HIV risk exposures including heterosexual and perinatal transmission. Illicit drug use was considered active if drug use occurred during pregnancy and included the use of amphetamines, barbiturates, benzodiazepines, cocaine, crack cocaine, heroin, hallucinogens, methadone, marijuana, methamphetamines, opiates, or other illegal drugs.

Mode of delivery was evaluated by the dichotomous maternal VL variable. The VL variable reflected the VL during pregnancy, closest to the time of delivery and was classified as VL ≤1,000 copies/ml vs. unknown VL or a VL >1,000 copies/ml. Women lacking a recorded VL at delivery are considered to be at greater risk for MTCT compared to women with a VL ≤1,000 and were therefore included with women with a VL >1,000 copies/ml. Other clinical variables of interest included history of a previous C-section delivery, quality of prenatal care as measured by the Kessner Index, ART receipt (at any point during pregnancy), and timing of ART initiation. The Kessner Index, a validated measure of adequacy of prenatal care which takes into account the month in which prenatal care began, gestational weeks at time of delivery and number of prenatal care visits, classifies care as adequate, intermediate or inadequate [13]. Classification as adequate indicates that the initial prenatal visit occurred within the first trimester (at or before 13 weeks) and a minimum of 9 prenatal care visits was attended if delivery occurred after 35 weeks’ gestation. Classification as inadequate/no prenatal care indicates that the initial prenatal care visit occurred in the third trimester (after 27 weeks) or fewer than 5 visits were after 18 weeks’ gestation. All other combinations of prenatal care initiation, number of prenatal care visits, and gestational age of delivery are considered intermediate. ART receipt during pregnancy was determined by provider documentation during the prenatal visits and hospitalizations; additional information on type or appropriateness of antiretroviral used was available but often incomplete. Timing of ART initiation during pregnancy was classified as ART initiation by the first or second trimester as opposed to ART initiated within the third trimester. Deliveries were also categorized by hospital site. Timing of HIV diagnosis was categorized as prior to pregnancy, during pregnancy, and at or soon after delivery based on when the HIV diagnosis was made.

Infant level variables included birth type, gestational age and HIV status. Birth type was categorized as single or multiple gestations. Since CDC and ACOG guidelines stipulate that a scheduled C-section for the purpose of minimizing vertical HIV transmission should be at 38 weeks’ gestation, the gestational age variable was classified as <38 weeks’ gestation or ≥38 weeks’ gestation [6–7]. Infant HIV status was determined using the CDC case definition for HIV-exposed infants under 18 months of age. HIV positivity required a positive result on two separate specimens (not including cord blood) from one or more HIV virologic tests (HIV nucleic acid detection, HIV antigen test, or HIV isolation)[14].

### Statistical Analysis

We examined maternal demographic, behavioral and clinical/engagement in care characteristics based on the mode of delivery and stratified by maternal viral load during pregnancy, closest to the time of delivery from 2005 to 2013. The relationship between infant characteristics such as birth type (single/multiple), infant HIV status and gestational age at birth and mode of delivery was also analyzed using univariable analysis. A multivariable logistic regression model was used to determine the odds of receiving a scheduled C-section when the VL was unknown or >1,000 copies/ml adjusting for demographic, clinical and engagement in care variables, delivery hospital site variable (data not shown), and clustering at the level of the mother due to repeated births during the study period. We hypothesized that non-Hispanic black women were more likely to deliver preterm prior to 38 weeks’ gestation and an additional multivariable analysis was conducted to adjust for an interaction between gestational age and race/ethnicity. Secondary analysis included a sensitivity analysis excluding women without a VL during pregnancy. In addition, we evaluated the proportion of women receiving scheduled C-sections after 38 weeks’ gestation with a third trimester VL >1,000 copies/mL and associations between scheduled C-section and timing of HIV diagnosis, timing of ART initiation, and mode of delivery. Statistical analyses were conducted using STATA 12 (StataCorp, College Station, TX).

## Results

### Study Population

Our cohort included 824 deliveries from 648 HIV-infected women of whom 51.3% were between 25–34 years of age, 79.0% were non-Hispanic black, and 84.8% had an HIV risk factor other than injection drug use. Of the total study population, 39.1% received adequate, 38.3% intermediate, and 22.6% inadequate prenatal care (Table 1). The majority of women (74.8%) were diagnosed prior to pregnancy, 172 (20.9%) during pregnancy, and 36 (4.4%) at time of delivery or soon after delivery. ART was prescribed during the majority of pregnancies (85.3%) and typically initiated by the first or second trimester (64.7%).

**Table 1:** Mode of Delivery among HIV-Infected Women with a Viral Load ≤1,000 and Unknown VL or VL >1,000 copies/ml by Demographic, Behavioral and Clinical Characteristics, 2005–2013—Enhanced Perinatal Surveillance, Philadelphia.^a^

		Viral Load ≤1000 copies/ml (n = 572)				Viral Load >1000 copies/ml or unknown (n = 252)			
	Total Population (n = 824)	Scheduled C-section	Emergent C-section	Vaginal Delivery	*p*-value	Scheduled C-section	Emergent C-section	Vaginal Delivery	*p*-value
**Total, n (%)**		172 (30.1)	76 (13.3)	324 (56.6)		125 (49.9)	44 (17.5)	83 (32.9)	
**Age**					**0.006**				0.257
16–24	211 (25.6)	37 (25.0)	19 (12.8)	92 (62.2)		38 (60.3)	10 (15.9)	15 (23.8)	
25–34	423 (51.3)	82 (27.3)	39 (13.0)	179 (59.7)		59 (48.0)	23 (18.7)	41 (33.3)	
≥ 35	190 (23.1)	53 (42.7)	18 (14.5)	53 (42.7)		28 (42.4)	11 (16.7)	27 (40.9)	
**Race**					0.153				0.055
White, Non-Hispanic	105 (12.7)	27 (40.9)	9 (13.6)	30 (45.5)		27 (69.2)	2 (5.1)	10 (25.6)	
Black, Non-Hispanic	651 (79.0)	129 (27.9)	60 (13.0)	273 (59.1)		89 (47.1)	36 (19.1)	64 (33.9)	
Hispanic	68 (8.3)	16 (36.4)	7 (15.9)	21 (47.7)		9 (37.5)	6 (25.0)	9 (37.5)	
**Marital Status**					0.826				0.643
Unmarried	670 (81.3)	134 (29.7)	59 (13.1)	259 (57.3)		107 (49.1)	40 (18.4)	71 (32.6)	
Married	154 (18.7)	38 (31.7)	17 (14.2)	65 (54.2)		18 (52.9)	4 (11.8)	12 (35.3)	
**Insurance Coverage**					0.729				0.733
Public	642 (77.9)	134 (31.0)	54 (12.5)	244 (56.5)		107 (51.0)	34 (16.2)	69 (32.9)	
Private	94 (11.4)	21 (28.4)	10 (13.5)	43 (58.1)		9 (45.0)	4 (20.0)	7 (35.0)	
Uninsured	88 (10.7)	17 (25.8)	12 (18.2)	37 (56.1)		9 (40.9)	6 (27.3)	7 (31.8)	
**HIV Risk Factor**					**0.004**				0.968
Other Risk^b^	699 (84.8)	137 (27.6)	68 (13.7)	292 (58.8)		101 (50.0)	35 (17.3)	66 (32.7)	
Injection Drug Use	125 (15.2)	35 (46.7)	8 (10.7)	32 (42.7)		24 (48.0)	9 (18.0)	17 (34.0)	
**Drug Use During Pregnancy**					0.256				0.116
No	635 (77.1)	137 (29.4)	58 (12.5)	271 (58.2)		91 (53.9)	29 (17.2)	49 (29.0)	
Yes	189 (22.9)	35 (33.0)	18 (17.0)	53 (50.0)		34 (41.0)	15 (18.1)	34 (41.0)	
**Previous Cesarean Delivery**					**<0.001**				**<0.001**
No	673 (81.7)	77 (16.2)	73 (15.4)	324 (68.4)		76 (38.2)	40 (20.1)	83 (41.7)	
Yes	151 (18.3)	95 (96.9)	3 (3.1)	0 (0.0)		49 (92.5)	4 (7.6)	0 (0.0)	
**Quality of Prenatal Care**					0.899				**0.004**
Adequate	322 (39.1)	73 (28.5)	37 (14.5)	146 (57)		41 (62.1)	6 (9.1)	19 (28.8)	
Intermediate	316 (38.3)	74 (32)	28 (12.1)	129 (55.8)		44 (51.8)	21 (24.7)	20 (23.5)	
Inadequate	186 (22.6)	25 (29.4)	11 (12.9)	49 (57.7)		40 (39.6)	17 (16.8)	44 (43.6)	
**ART Prescription During Pregnancy**					0.649				**<0.001**
No	121 (14.7)	5 (21.7)	3 (13.0)	15 (65.2)		33 (33.7)	14 (14.3)	51 (52.0)	
Yes	703 (85.3)	167 (30.4)	73 (13.3)	309 (56.3)		92 (59.7)	30 (19.5)	32 (20.8)	
**ART Initiation by 1st/2nd Trimester**					0.764				**<0.001**
No	291 (35.3)	42 (27.8)	20 (13.3)	89 (58.9)		56 (40.0)	25 (17.9)	59 (42.1)	
Yes	533 (64.7)	130 (30.9)	56 (13.3)	235 (55.8)		69 (61.6)	19 (17.0)	24 (21.4)	
**Time of HIV Diagnosis**					0.337				**0.004**
Prior to Pregnancy	616 (74.8)	140 (31.1)	56 (12.4)	254 (56.4)		90 (54.2)	25 (15.1)	51 (30.7)	
During Pregnancy	172 (20.9)	29 (25.0)	19 (16.4)	68 (58.6)		30 (53.6)	11 (19.6)	15 (26.8)	
At/After Delivery	36 (4.4)	3 (50.0)	1 (16.7)	2 (3.33)		5 (16.7)	8 (26.7)	17 (56.7)	
**Gestational Age at Delivery**					0.088				**<0.001**
< 38 weeks	260 (31.6)	47 (29.6)	29 (18.2)	83 (52.2)		35 (34.7)	31 (30.7)	35 (34.7)	
≥ 38 weeks	564 (68.5)	125 (30.3)	47 (11.4)	241 (58.4)		90 (59.6)	13 (8.6)	48 (31.8)	
**Birth Type**					**0.013**				0.157
Single	803 (97.5)	165 (29.7)	71 (12.8)	320 (57.6)		122 (49.4)	42 (17.0)	83 (33.6)	
Multiple	21 (2.5)	7 (43.8)	5 (31.3)	4 (25.0)		3 (60.0)	2 (40.0)	0 (0.0)	
**Infant's HIV Status**					0.626				0.07
HIV Positive	16 (1.9)	2 (50.0)	1 (25.0)	1 (25.0)		2 (16.7)	2 (16.7)	8 (66.7)	
HIV Negative	658 (79.9)	139 (30.1)	60 (13.0)	263 (56.9)		104 (53.1)	33 (16.8)	59 (30.1)	
Indeterminate	150 (18.2)	31 (29.3)	15 (14.2)	60 (56.6)		19 (43.2)	9 (20.5)	16 (36.4)	

Abbreviation: *ART*, Antiretroviral Therapy

^a^ Infants of a multiple gestation were counted as one case. Analyses were restricted to women without an unknown/missing documentation of mode of delivery. Delivery hospital information was not included for confidentiality purposes.

^b^ Other Risk included heterosexual and perinatal transmissions.

*P*-values are derived from Chi-square and Fisher’s Exact Test. Boldface font, p < 0.05

### Mode of delivery overall and by maternal HIV viral load

Of the total study population of 824 deliveries, 407 pregnancies (49.4%) resulted in vaginal deliveries, 297 (36.0%) in scheduled C-sections and 120 (14.6%) in emergent C-sections. In univariable analysis stratified by maternal VL, for women with a VL ≤1,000 copies/ml, mode of delivery differed by maternal age, HIV risk factor, previous C-section, and birth type; for women with a VL >1,000 copies/ml, mode of delivery differed by previous C-section, quality of prenatal care, ART prescription during pregnancy, time of HIV diagnosis, and gestational age at delivery. (Table 1).

Close to thirty percent of the cohort (n = 252, 30.6%) had an unknown VL or a VL >1,000 copies/ml during pregnancy, closest to the time of delivery while 69.4% (n = 572) had a VL ≤1,000 copies/ml. Out of 252 deliveries with VL >1,000 copies/ml or unknown VL, 49.9% had a scheduled C-section, 32.9% a vaginal delivery, and 17.5% an emergent C-section (Table 1). In comparison, out of 572 deliveries with an VL ≤1,000 copies/ml, 30.1% had a scheduled C-section, 56.6% had a vaginal delivery, and 13.3% had an emergent C-section. Overall, 31.6% of the cohort delivered before 38 weeks’ gestation. Women without a VL or VL >1,000 copies/ml and delivering before 38 weeks’ gestation were less likely to deliver via scheduled C-section (34.7% vs. 59.6%, p-value <0.001) and more likely to deliver via emergent C-section (30.7% vs. 8.6%, p-value <0.001) compared to women delivering at or after 38 weeks’ gestation. ART was initiated by the first or second trimester for 73.6% (n = 421/572) of women with a VL ≤1,000 copies/mL compared to 44.4% (n = 112/252) of women with a VL >1,000 copies/mL or without a VL during pregnancy (p-value <0.001).

Of 824 HIV-exposed infants, 16 (1.9%) were confirmed HIV-infected. Seventy-five percent (n = 12) of the HIV-infected infants were born to women without a VL or a VL >1,000 copies/ml during pregnancy, closest to the time of delivery. Of the 12 HIV-infected infants born to women without a VL or a VL>1,000 copies/ml at delivery, 8 were born via vaginal delivery, 2 via emergent C-section, and 2 via a scheduled C-section.

### Maternal Characteristics by Gestational Age at Delivery

A substantial portion of our cohort (n = 260, 31.6%) delivered before 38 weeks’ gestation (Table 2). Within univariable analysis, women who used drugs were more likely to deliver <38 weeks’ gestation compared to women who did not use drugs (41.8% vs. 22.9%), less likely to have received adequate prenatal care (25.2% vs. 39.1%), less likely to have an ART prescription during pregnancy (29.4% vs. 85.3%), and less likely to have initiated ART by the first or second trimester (27.2% vs. 64.7%). While not statistically significant in univariable analysis, a larger proportion of non-Hispanic black (31.5%) and Hispanic (38.2%) women delivered before 38 weeks’ gestation compared to non-Hispanic white women (27.6%).

**Table 2:** Maternal characteristics among HIV-infected pregnant women by gestational age, Philadelphia, 2005–2013.

	<38 Weeks' Gestation at Time of Delivery (n = 260)	≥38 Weeks' Gestation at Time of Delivery (n = 564)	*p*-value
**Age at Delivery (yr)**			0.082
16–24	55 (26.1)	156 (73.9)	
25–34	136 (32.2)	287 (67.8)	
≥35	69 (36.3)	121 (63.7)	
**Race/Ethnicity**			0.34
White, Non-Hispanic	29 (27.6)	76 (72.4)	
Black, Non-Hispanic	205 (31.5)	446 (68.5)	
Hispanic	26 (38.2)	42 (61.8)	
**Marital Status**			0.787
Unmarried	210 (31.3)	460 (68.7)	
Married	50 (32.5)	104 (67.5)	
**Insurance Coverage**			0.08
Public	215 (33.5)	427 (66.5)	
Private	23 (24.5)	71 (75.5)	
Uninsured	22 (25.0)	66 (75.0)	
**HIV Risk Factor**			0.114
Other Risk^a^	213 (30.5)	486 (69.5)	
Injection Drug Use	47 (37.6)	78 (62.4)	
**Drug Use During Pregnancy**			**0.001**
No	181 (28.5)	454 (71.5)	
Yes	79 (41.8)	110 (58.2)	
**Previous Cesarean Delivery**			0.062
No	222 (33.0)	451 (67.0)	
Yes	38 (25.2)	113 (74.8)	
**Quality of Prenatal Care**			**0.001**
Adequate	81 (25.2)	241 (74.8)	
Intermediate	103 (32.6)	213 (67.4)	
Inadequate	76 (40.9)	110 (59.1)	
**ART Prescription During Pregnancy**			**0.002**
No	53 (43.8)	68 (56.2)	
Yes	207 (29.4)	496 (70.6)	
**ART Initiated By 1st/2nd Trimester**			**<0.001**
No	115 (39.5)	176 (60.5)	
Yes	145 (27.2)	388 (72.8)	
**Time of HIV Diagnosis**			
Prior to Pregnancy	189 (30.7)	427 (69.3)	0.116
During Pregnancy	54 (31.4)	118 (68.6)	
At/After Time of Delivery	17 (47.2)	19 (52.8)	

^a^ Other Risk included heterosexual and perinatal transmissions.

### Maternal factors associated with a scheduled C-section among women with a VL>1,000 copies/ml

In univariable analyses, we found that non-Hispanic black women (Unadjusted Odds Ratio (UOR) 0.38, 95% CI 0.17–0.83) and Hispanic women (UOR 0.25, 95% CI 0.09–0.76) were less likely to receive a scheduled C-section compared to non-Hispanic white women (Table 3). Women over the age of 35 years old (UOR 0.48, 95% CI 0.24–0.97) were also less likely to receive a scheduled C-section compared to younger women. Women with a previous C-section (UOR 19.80, 95% CI 6.85–56.98) and those prescribed ART during pregnancy (UOR 2.97, 95% CL 1.73–5.10) were more likely to deliver via C-section than their counterparts. Women who delivered prior to 38 weeks’ gestation were less likely to deliver by scheduled C-section (UOR 0.36, 95% CI 0.21–0.61) compared to those with term deliveries. Women who were diagnosed with HIV at or soon after delivery (UOR 0.17, 95% CI 0.06–0.46) and women who received inadequate prenatal care (UOR 0.40, 95% CI 0.21–0.75) were also less likely to deliver by scheduled C-section compared to women who were diagnosed prior to pregnancy and women who received adequate prenatal care. Women prescribed ART during pregnancy (UOR 2.97, 95% CI 1.44–5.10) and initiating ART by the first or second trimester (UOR 2.43, 95% CI 1.44–4.09) were more likely to deliver via scheduled C-section compared to women who had not initiated ART by the first or second trimester or women not prescribed ART during pregnancy in unadjusted analysis.

**Table 3:** Factors Associated with Delivery via Scheduled C-Section among HIV-Infected Women with an Unknown Viral Load or Viral Load >1,000 copies/ml (n = 321), Philadelphia, 2005–2013.

	UOR (95% CI)	*p*-value	AOR (95% CI)	*p*-value	AOR (95% CI) with Interaction	*p*-value
**Age at Delivery (yr)**						
16–24	1 [Reference]		1 [Reference]		1 [Reference]	
25–34	0.61 (0.33–1.10)	0.099	0.62 (0.28–1.38)	0.256	0.56 (0.24–1.31)	0.178
≥35	0.48 (0.24–0.97)	**0.042**	0.42 (0.17–1.02)	0.056	0.39 (0.15–1)	**0.050**
**Race/Ethnicity**						
White, Non-Hispanic	1 [Reference]		1 [Reference]		1 [Reference]	
Black, Non-Hispanic	0.38 (0.17–0.83)	**0.016**	0.27 (0.10–0.77)	**0.014**	0.14 (0.05–0.41)	**<0.001**
Hispanic	0.25 (0.09–0.76)	**0.014**	0.17 (0.04–0.76)	**0.021**	0.08 (0.01–0.47)	**0.005**
**Marital Status**						
Unmarried	1 [Reference]		1 [Reference]		1 [Reference]	
Married	1.16 (0.55–2.43)	0.690	0.39 (0.15–1.07)	0.068	0.36 (0.13–1.01)	0.051
**Insurance Coverage**						
Public	1 [Reference]		1 [Reference]		1 [Reference]	
Private	0.79 (0.31–2.07)	0.639	0.83 (0.20–3.42)	0.792	0.89 (0.21–3.72)	0.868
Uninsured	0.67 (0.29–1.56)	0.355	0.54 (0.11–2.49)	0.431	0.48 (0.09–2.61)	0.394
**HIV Risk Factor**						
Other Risk	1 [Reference]		1 [Reference]		1 [Reference]	
Injection Drug Use	0.93 (0.50–1.74)	0.819	1.17 (0.49–2.83)	0.720	1.13 (0.45–2.83)	0.792
**Drug Use During Pregnancy**						
No	1 [Reference]		1 [Reference]		1 [Reference]	
Yes	0.60 (0.34–1.03)	0.065	0.68 (0.28–1.64)	0.388	0.78 (0.3–1.98)	0.594
**Previous Caesarean Delivery**						
No	1 [Reference]		1 [Reference]		1 [Reference]	
Yes	19.8 (6.85–56.98)	**<0.001**	27.77 (8.94–86.18)	**<0.001**	31.38 (9.44–104.29)	**<0.001**
**Quality of Prenatal Care**						
Adequate	1 [Reference]		1 [Reference]		1 [Reference]	
Intermediate	0.65 (0.34–1.25)	0.199	0.60 (0.27–1.35)	0.221	0.62 (0.26–1.44)	0.267
Inadequate	0.40 (0.21–0.75)	**0.004**	0.46 (0.15–1.38)	0.165	0.46 (0.14–1.49)	0.195
**ART Prescription during Pregnancy**						
No	1 [Reference]		1 [Reference]		1 [Reference]	
Yes	2.97 (1.73–5.10)	**<0.001**	1.25 (0.40–3.85)	0.696	1.24 (0.38–4.1)	0.722
**ART initiated by 1st/2nd trimester**						
No	1 [Reference]		1[Reference]		1 [Reference]	
Yes	2.43 (1.44–4.09)	**<0.001**	1.12 (0.41–3.11)	0.821	1.43 (0.50–4.12)	0.507
**Gestational Age at Time of Delivery**						
<38 weeks	0.36 (0.21–0.61)	**<0.001**	0.37 (0.18–0.76)	**0.007**	0.42 (0.13–1.37)	0.149
≥38 weeks	1 [Reference]		1 [Reference]		1 [Reference]	
**Time of HIV Diagnosis**						
Prior to Pregnancy	1 [Reference]		1 [Reference]		1 [Reference]	
During Pregnancy	0.97 (0.54–1.76)	0.931	1.70 (0.78–3.73)	0.188	1.89 (0.79–4.48)	0.151
At/After Delivery	0.17 (0.06–0.46)	**<0.001**	0.32 (0.06–1.67)	0.177	0.35 (0.06–2.09)	0.248
**Race/Ethnicity x Gestational Age at Time of Delivery**						
Black and <38 weeks	-	**-**	-		0.06 (0.01–0.36)	**0.002**
Hispanic and < 38 weeks	-	**-**	-		0.03 (0.00–0.59)	**0.021**

Delivery hospital information was not included for confidentiality purposes.

*AOR*, adjusted odds ratio; *CI*, confidence interval; *UOR*, unadjusted odds ratio; *bold face font*, p <0.05.

In multivariable analyses, race, gestational age at delivery, and history of a previous C-section remained significant. Women who delivered prior to 38 weeks’ gestation (0.37, 95% CI 0.18–0.76), Hispanic (AOR 0.17, 95% CI 0.04–0.76), and non-Hispanic black women (AOR 0.27, 95% CI 0.10–0.77) were less likely to deliver via scheduled C-section compared to women delivering at or after 38 weeks’ gestation and non-Hispanic white women. Women with a previous C-section (AOR 27.77, 95% CI 8.94–86.18) were also more likely to have a C-section delivery than women with no prior C-sections. Women delivered in eight hospitals, mostly university affiliated, located in Philadelphia and surrounding areas. There was no statistically significant difference in the prevalence of scheduled C-sections and VL distribution at delivery by delivery hospital (data not shown).

Addition of a race/ethnicity and gestational age interaction term resulted in effect modification within the multivariable logistic regression model. Independently, age became newly significant and race remained significantly associated with receipt of a scheduled C-section. In addition, the interaction term was statistically significant; suggesting the association between race and a scheduled C-section varies by gestational age at the time of delivery. Non-Hispanic black women (AOR 0.06, 95% CI 0.01–0.36) and Hispanic women (AOR 0.03, 95% CI 0.00–0.59) who delivered <38 weeks’ gestation were less likely to have a scheduled C-section when VL was unknown or >1,000 copies/ml during pregnancy, closest to the time of delivery. Of the women with an unknown VL or VL >1,000 copies/ml who delivered prior to 38 weeks’ gestation, 75.0% (n = 9/12) of white women delivered via scheduled C-section compared to 30.8% (n = 24/78) of non-Hispanic black and 18.2% (n = 2/11) Hispanic women (data not shown). History of a previous C-section remained significant. A sensitivity analysis excluding unknown VL (n = 84) showed no statistically significant difference in study findings.

### HIV-infected women with VL >1,000 copies/ml in third trimester

Viral load information during the third trimester was available for the majority of our study population. For women with a VL >1,000 copies/ml in the third trimester (n = 130), 49 (37.7%) delivered before 38 weeks’ gestation. For the remaining 81 women, 74.1% had a scheduled C-section, 8.6% an emergent C-section, and 17.3% a vaginal delivery. Of women delivering after 38 weeks’ gestation with a third trimester VL >1,000, 55.6% had initiated ART by the second trimester and 72.8% had been diagnosed with HIV prior to pregnancy.

## Discussion

In this cohort of HIV-infected pregnant women in Philadelphia, guidelines recommending a scheduled C-section for women with a VL >1,000 copies/ml were not consistently met. Our findings identified multiple missed opportunities for HIV-infected pregnant women including a high rate of premature delivery (<38 weeks’ gestation) particularly among women of color, lack of viral load information and viral suppression during pregnancy, and the failure to schedule C-sections for women with a VL > 1,000 copies/ml.

While the majority of women delivered after 38 weeks’ gestation, Hispanic and non-Hispanic black women were significantly more likely to deliver prior to 38 weeks’ gestation compared to non-Hispanic white women. Premature delivery contributed to significant racial disparities: non-Hispanic black and Hispanic women were over 70% less likely to receive a scheduled C-section when indicated compared to non-Hispanic white women even after controlling for demographic, clinical, and engagement in care variables. Nationally, the percentage of early term (37 0/7-38 6/7 weeks' gestation) deliveries via scheduled C-section without medical indication has increased 86% from 1995 to 2009, mostly among white, insured, and college educated women [9]. Within our study, women of color were more likely to deliver prior to 38 weeks’ gestation and less likely to deliver via scheduled C-section compared to non-Hispanic white women. According to national data, the proportion of preterm deliveries is higher among black women compared to white women (16.53% vs. 10.29%, respectively) [15]. Women delivering prematurely often present with ruptured membranes, increasing the likelihood of MTCT [1]. This clinical finding calls for improved surveillance and interventions to reduce the risk of prematurity, particularly among women of color. Univariable analyses showed that women with inadequate prenatal care, prenatal drug use, and no or late ART use during pregnancy were also more likely to deliver prematurely (Table 2). An association between prenatal substance use and preterm delivery has been previously reported [16]. Offering concurrent substance abuse treatment with prenatal care has been shown to decrease the likelihood of preterm delivery and may be applicable to HIV-infected women with prenatal substance use [16]. Measures to reduce the risk of premature labor, including early and continued prenatal care, should be emphasized, particularly among women with elevated VLs at presentation for care. Preventative interventions such as weekly progesterone injections or the use of tocolytic agents have been applied with varying degrees of success in the general population [17, 18]; however, tools to identify pregnant women for whom these and other interventions will effectively reduce preterm birth are lacking. Of note, C-section deliveries scheduled before 38 weeks’ gestation may increase associated costs and infant complications [19]. Within our study, non-Hispanic white women delivering prior to 38 weeks’ gestation were more likely to deliver via scheduled C-section compared to non-Hispanic black and Hispanic women. However, the reduced risk of vertical transmission associated with scheduled C-sections before 38 weeks’ may not offset the risk of maternal and infant morbidity as MTCT in our cohort was less than the national average (2%) even with moderate compliance to current recommendations. Early prenatal care and timely initiation of ART during pregnancy are better candidates for improving maternal and fetal outcomes.

A large proportion of our cohort had an unknown VL or a VL >1,000 copies/ml near delivery, calling for increased attention to guidelines for virologic testing as well as for achieving viral suppression during pregnancy. The majority of our cohort (85.3%) received ART during pregnancy; slightly more than the national EPS average (84%) and other reported frequencies in U.S. and international studies [11, 20]. However, virologic control was still quite low: only 69.4% of women had a VL ≤1,000 copies/ml during pregnancy, closest to the time of delivery, suggesting that complete viral suppression was even lower. According to a 2011 estimate from the CDC, 77% of HIV-infected adults prescribed ART achieve viral suppression (HIV VL≤ 200 copies/ml) [21]. Of note, our study involved women at various levels of the HIV care continuum, including women newly diagnosed with HIV. Typically a pregnant woman has multiple interactions with the healthcare system within a short period of time. Prenatal care visits offer opportunities to discuss the initiation of and adherence to ART. Qualitative analyses have shown that HIV-infected pregnant women have a high level of motivation to be adherent to ART in order to prevent MTCT of HIV compared to non-pregnant women; these findings support the fact that viral suppression by delivery is an achievable goal if psychosocial and structural barriers to ART adherence are addressed early in a woman’s pregnancy [22]. Viral suppression at delivery is very likely when ART is initiated before 20 weeks’ gestation and is possible with appropriate treatment even much later in pregnancy [23]. However, socioeconomic challenges, stigma, isolation, mental health and substance abuse [24, 25] can interfere with ART adherence in this critical period of women’s lives and these factors may have contributed to our findings. Tools are needed to identify barriers to ART adherence along with interventions to link and retain pregnant HIV-infected women in care. Interventions for the general HIV population such as case management [26] and patient navigator [27] involvement have successfully increased linkage, retention, and viral suppression and could improve clinical outcomes for HIV-infected women during pregnancy and postpartum.

Even among women delivering at or after 38 weeks’ gestation, 25% with a VL >1,000 copies/ml did not receive a scheduled C-section. This finding calls for efforts to increase the proportion of women delivering via Caesarean when indicated. The substantial effectiveness of a scheduled C-section delivery in reducing MTCT of HIV in the pre-combination (cART) era and post-cART era is well established [2, 4]. While we were unable to detect a difference in surgical practices based on delivery hospital, patient preferences and/or provider perceptions or bias may have contributed to the low prevalence of scheduled C-sections. Studies have shown that physician factors such as gender, financial incentives, convenience, and education/training influences the percentage of women receiving C-sections nationally [28–32]. However, financial reimbursement is an unlikely driver of our findings as type of insurance coverage was not associated with receipt of a scheduled C-section among HIV-infected women with a VL >1,000 copies/mL in either univariable or multivariable analysis.

Our study has several limitations. Relying on surveillance data obtained mainly from chart abstractions revealed errors associated with missing values, misclassification, and under-reporting of data. Some women lacked a VL during pregnancy; however, a sensitivity analysis was performed to ensure that unknown VL at delivery during pregnancy closest to the time of delivery did not impact major study findings. Secondly, patients or providers were not contacted to understand factors associated with mode of delivery. Future qualitative analyses are needed to further evaluate these factors. EPS abstraction forms did not collect data on whether a C-section was scheduled and not performed but discerning this information would be helpful for future studies. The data are also limited to a single geographic region and may not be applicable to the general population of HIV-infected pregnant women. Finally, the use of the Kessner index limited the interpretation of the adequacy of prenatal care to only one index. Results may have been altered slightly if other tools for measuring adequacy were used. Despite limitations, the EPS system provided ongoing surveillance and data abstracts from various sources over a long period of time.

In conclusion, only half of deliveries for women with an unknown VL or VL >1,000 copies/ml occurred via scheduled C-section. Delivery prior to 38 weeks, particularly among minority women, resulted in a missed opportunity to receive a scheduled C-section. However, even when delivering at or after 38 weeks’ gestation, a quarter of the women with a VL >1,000 copies/ml had a vaginal delivery or emergent C-section, suggesting a need for focused public health interventions to increase the proportion of women achieving viral suppression during pregnancy and delivering via scheduled C-section when indicated.

## References

[1] Suy A, Hernandez S, Thorne C, Lonca M, Lopez M, Coll O. Current guidelines on management of HIV-infected pregnant women: Impact on mode of delivery. Eur J Obstet Gynecol Reprod Biol. 2008; 139(2):127–132. 10.1016/j.ejogrb.2007.12.007 18262324

[2] Momplaisir FM, Brady KA, Fekete T, Thompson DR, Diez Roux A, Yehia BR. Time of HIV diagnosis and engagement in prenatal care impact virologic outcomes of pregnant women with HIV. PLoS ONE. 2015; 10(7): e0132262 doi: 10.1371/ journal.pone.0132262 26132142 10.1371/journal.pone.0132262 PMC4489492

[3] Garcia PM, Kalish LA, Pitt J, Minkoff H, Quinn TC, Burchett SK, et al Maternal levels of plasma human immunodeficiency virus type 1 RNA and the risk of perinatal transmission. N Engl J Med. 1999; 341(6):394–402. 10432324 10.1056/NEJM199908053410602

[4] Parazzini F, Ricci E, Di Cintio E, Chiaffarino F, Chatenoud L, Pardi G, et al Elective caesarean-section versus vaginal delivery in prevention of vertical HIV-1 transmission: a randomised clinical trial. Lancet. 1999; 353(9158):1035–1039. 10199349 10.1016/s0140-6736(98)08084-2

[5] Read J, Scherpbier H, Boer K. The mode of delivery and the risk of vertical transmission of human immunodeficiency virus type 1: a meta-analysis of 15 prospective cohort studies: the International Perinatal HIV Group. N Engl J Med. 1999; 340:977–987. 10099139 10.1056/NEJM199904013401301

[6] Practice CoO. ACOG committee opinion scheduled Cesarean delivery and the prevention of vertical transmission of HIV infection. Number 234, May 2000 (replaces number 219, August 1999). Int J Gynaecol Obstet. 2001; 73(3):279 11424912 10.1016/s0020-7292(01)00412-x

[7] Panel on Treatment of HIV-Infected Pregnant Women and Prevention of Perinatal Transmission. Recommendations for Use of Antiretroviral Drugs in Pregnant HIV-1-Infected Women for Maternal Health and Interventions to Reduce Perinatal HIV Transmission in the United States. 2015. Available: https://aidsinfo.nih.gov/contentfiles/lvguidelines/perinatalgl.pdf.

[8] Short CE, Douglas M, Smith J, Taylor G. Preterm delivery risk in women initiating antiretroviral therapy to prevent HIV mother-to-child transmission. HIV Med. 2014; 15(4):233–238. 10.1111/hiv.12083 24025074

[9] Kozhimannil KB, Macheras M, Lorch SA. Trends in Childbirth before 39 Weeks’ Gestation without Medical Indication. Med Care. 2014; 52(7):649–657. 10.1097/MLR.0000000000000153 24926713 PMC4096132

[10] Béhague DP, Victora CG, Barros FC. Consumer demand for caesarean sections in Brazil: informed decision making, patient choice, or social inequality? A population based birth cohort study linking ethnographic and epidemiological methods. BMJ. 2002; 324(7343):942 11964338 10.1136/bmj.324.7343.942 PMC102326

[11] Centers for Disease Control and Prevention. Enhanced perinatal surveillance—15 areas, 2005–2008. HIV Surveillance Supplemental Report 2011. In. http://www.cdc.gov/hiv/topics/surveillance/resources/reports/.

[12] Centers for Disease Control and Prevention. HIV Surveillance Report, 2013. 2015; 25. Available: http://www.cdc.gov/hiv/library/reports/surveillance/.

[13] Kotelchuck M. An evaluation of the Kessner adequacy of prenatal care index and a proposed adequacy of prenatal care utilization index. Am J Public Health. 1994; 84(9):1414–1420. 8092364 10.2105/ajph.84.9.1414 PMC1615177

[14] Centers for Disease Control and Prevention. Revised surveillance case definitions for HIV infection among adults, adolescents, and children aged <18 Months and for HIV infection and AIDS among children aged 18 months to <13 years. MMWR 2008; 57(No. RR-10). 19052530

[15] Martin JA, Hamilton BE, Ventura SJ, Osterman MJ, Matthews T. Births: final data for 2011. National Vital Statistics Report. 2013; 62(1). 24974591

[16] Goler NC, Armstrong MA, Taillac CJ Osejo VM. Substance abuse treatment linked with prenatal visits improves perinatal outcomes: A new standard. Journal of Perinatology. 2008; 28 (9): 597–603. 10.1038/jp.2008.70 18580882

[17] Meis PJ, Klebanoff M, Thom E, Dombrowski MP, Sibai B, Moawad AH, et al Prevention of recurrent preterm delivery by 17 alpha-hydroxyprogesterone caproate. N Engl J Med. 2003; 348(24):2379–2385. 12802023 10.1056/NEJMoa035140

[18] Simhan HN, Caritis SN. Prevention of preterm delivery. N Engl J Med. 2007; 357(5):477–487. 17671256 10.1056/NEJMra050435

[19] Villar J, Carroli G, Zavaleta N, Donner A, Wojdyla D, Faundes A, et al Maternal and neonatal individual risks and benefits associated with caesarean delivery: multicentre prospective study. BMJ. 2007; 335(7628):1025 17977819 10.1136/bmj.39363.706956.55 PMC2078636

[20] Wettstein C, Mugglin C, Egger M, Blaser N, Vizcaya LS, Estill J, et al Missed opportunities to prevent mother-to-child-transmission: systematic review and meta-analysis. AIDS (London, England). 2012; 26(18):2361–2373. 10.1097/QAD.0b013e328359ab0c PMC3741537 22948267

[21] Centers_for_Disease_Control_Prevention. Vital signs: HIV prevention through care and treatment—United States. MMWR. Morbidity and mortality weekly report. 2011; 60(47):1618. 22129997

[22] Nachega JB, Uthman OA, Anderson J, Peltzer K, Wampold S, Cotton MF, et al Adherence to antiretroviral therapy during and after pregnancy in low-income, middle-income, and high-income countries: a systematic review and meta-analysis. AIDS. 2012; 26(16):2039–2052. 10.1097/QAD.0b013e328359590f 22951634 PMC5061936

[23] Aebi-Popp K, Mulcahy F, Glass TR, Rudin C, Martinez de Tejada B, Bertisch B, et al Missed opportunities among HIV-positive women to control viral replication during pregnancy and to have a vaginal delivery. J Acquir Immune Defic Syndr. 2013; 64(1):58–65. 10.1097/QAI.0b013e3182a334e3 23842074

[24] Turan JM, Nyblade L. HIV-related stigma as a barrier to achievement of global PMTCT and maternal health goals: a review of the evidence. AIDS Behav. 2013; 17(7):2528–2539. 10.1007/s10461-013-0446-8 23474643

[25] Myer L, Zulliger R, Bekker L-G, Abrams E. Systemic delays in the initiation of antiretroviral therapy during pregnancy do not improve outcomes of HIV-positive mothers: a cohort study. BMC Pregnancy Childbirth. 2012; 12(1):94. 22963318 10.1186/1471-2393-12-94 PMC3490939

[26] Gardner LI, Metsch LR, Anderson-Mahoney P, Loughlin AM, del Rio C, Strathdee S, et al Efficacy of a brief case management intervention to link recently diagnosed HIV-infected persons to care. AIDS. 2005; 19(4):423–431. 15750396 10.1097/01.aids.0000161772.51900.eb

[27] Bradford JB, Coleman S, Cunningham W. HIV System Navigation: an emerging model to improve HIV care access. AIDS Patient Care STDS. 2007; 21(S1): S-49–S-58. 17563290 10.1089/apc.2007.9987

[28] Mitler LK, Rizzo JA, Horwitz SM. Physician gender and cesarean sections. J Clin Epidemiol. 2000; 53(10):1030–1035. 11027936 10.1016/s0895-4356(00)00221-3

[29] Dale Tussing A, Wojtowycz MA. The effect of physician characteristics on clinical behavior: cesarean section in New York State. Soc Sci Med. 1993; 37(10):1251–1260. 8272903 10.1016/0277-9536(93)90336-3

[30] Burns LR, Geller SE, Wholey DR. The effect of physician factors on the cesarean section decision. Med Care. 1995; 33(4):365–382. 7731278 10.1097/00005650-199504000-00004

[31] Stafford RS. The impact of nonclinical factors on repeat cesarean section. JAMA. 1991; 265(1):59–63. 1984126

[32] Keeler EB, Brodie M. Economic incentives in the choice between vaginal delivery and cesarean section. The Milbank Quarterly. 1993:365–404. 8413067

